# Which Females Have the Highest Rates of Depression and Anxiety Following Acute Coronary Syndrome?

**DOI:** 10.1159/000528624

**Published:** 2022-12-15

**Authors:** David R. Thompson

**Affiliations:** School of Nursing and Midwifery, Queen's University Belfast, Belfast, UK

There is growing recognition of the impact of psychosocial factors such as depression and anxiety on cardiovascular disease, as exemplified in recent guidelines from the European Society of Cardiology [1] and a Scientific Statement from the American Heart Association [2]. There is also growing evidence from studies [3, 4, 5, 6] and reviews [7, 8] that following an acute coronary syndrome (ACS) women have significantly higher rates of depression and anxiety, which is associated with adverse outcomes such as increased morbidity, rehospitalization, mortality and decreased quality of life. For example, in the EUROASPIRE IV survey of 7,589 patients from 24 countries with coronary heart disease, symptoms of depression were seen in 31% of women and 20% of men, and symptoms of anxiety in 39% of women and 22% of men [4]. Depression, especially, and anxiety are associated with other risk factors, including lower levels of educational attainment, sedentary or inactive lifestyle, smoking, unhealthy diet and reduced adherence with lifestle changes or risk factor modification [4, 5].

Why are females at increased risk for depression and anxiety and which female patients have the highest rates? It is imperative that studies aim to understand why depression and anxiety disproportionately impact women following an ACS and how this knowledge can be used to better inform care.

In this issue of Cardiology, Liblik et al. [9] present the trial design for the Female Risk factors for post-Infarction Depression and Anxiety (FRIDA) Study which aims to identify which psychosocial and cardiovascular risk factors place female patients (as defined by sex) at highest risk of developing depressive and anxious symptoms following an ACS and to correlate these factors to mortality, morbidity, and quality of life outcomes at three and 6 months.

This questionnaire-based study [9], informed by a pilot study [10] and a narrative review [8] aims to recruit 20,000 female participants admitted to hospital with ACS will be conducted across seven sites in Canada, chosen to reflect a diversity of female patients, including racial, socioeconomic, cultural, and linguistic characteristics. Data pertaining to sociodemographic status, social support and depression and anxiety will be collected within 72 h of admission and at three and 6-month follow-up. Depression and anxiety will be measured using the Hospital Anxiety and Depression Scale (HADS), a well-validated and widely used questionnaire [11]. Additional follow-up data will include changes to health status and quality of life indicators. The primary aim is to determine if specific cardiovascular and sociodemographic factors correlate with increased depression and anxiety scores (HADS-D >7; HADS-A >7) at baseline. A secondary aim is to determine if increased HADS scores at baseline and follow-up correlate with three and 6-month health (major adverse cardiovascular events) and quality of life outcomes. Both aims are to be assessed by conventional statistical methods and machine learning classifications. Careful thought has been given to the analysis with machine learning methods being used to generate predictive insights from the data.

This is a well-designed, ambitious, and complex study. The authors appear to have given careful and detailed consideration of a host of factors and are cognisant of the challenges posed. For example, as they note, when conducting studies of this nature to assess the relationship between psychosocial determinants and health, establishing the link between sex and the psychosocial risks related to cardiovascular health is complex, and can be influenced by, for example, personal behaviour, lifestyle, and living/environmental conditions, economic context, genetic make-up, and, particular to females, exposure to other medical conditions such as a history of pre-eclampsia, contraception or hormonal treatments or changes. The authors have, therefore, quite sensibly and pragmatically, chosen to focus on mental health as representative of an important aspect of the convergence of these cultural, social, and environmental factors. This study will help by delineating those cardiovascular risk factors and social determinants of health which make specific groups of female patients more vulnerable to developing depressive and anxious symptoms following ACS. As the authors conclude, improving depressive and anxious symptoms in this population has the potential to reduce associated adverse cardiovascular outcomes.

The authors point out that it is important to note that while the HADS is a widely used instrument to evaluate depressive and anxious symptoms, it is not a diagnostic tool. However, it is widely used to evaluate depression and anxiety following an ACS. The authors also acknowledge other potential limitations. In addition, there are several important considerations that should be noted. First, it is unclear why the Cardiac Anxiety Questionnaire (CAQ) is being administered at three and 6-month follow-up, but not at baseline, in addition to the HADS. If the rationale is because the CAQ is a cardiac disease-specific measure, why not use a similar measure for depression, such as the Cardiac Depression Scale (CDS)? Second, why are two different measures of social support being used: the ENRICHD Social Support Inventory at baseline and the MOS Social Support Survey at follow-up? Third, why not measure health-related quality of life (Short Form-12 Health Survey) at baseline as well as at three and 6-month follow-up?

However, despite these, possibly pedantic, reservations, Liblik et al. [9] should be commended on designing such an important study, the results of which are eagerly awaited. The study will likely make a significant contribution to the literature and aid our understanding of the complexity of psychosocial factors potentially contributing to mental health and cardiovascular disease. Moreover, as it aims to establish which groups of female patients have the highest rates of depression and anxiety, it has the potential to inform prevention and intervention strategies for them.

## Conflict of Interest Statement

The author has no conflicts of interest to declare.

## Funding Sources

No funding was received for this editorial.

## Author Contributions

David R. Thompson wrote the editorial.
